# Total Endovascular Aortic Arch Repair: From Dream to Reality

**DOI:** 10.3390/medicina58030372

**Published:** 2022-03-02

**Authors:** Augusto D’Onofrio, Raphael Caraffa, Giorgia Cibin, Michele Antonello, Gino Gerosa

**Affiliations:** 1 Division of Cardiac Surgery, Department of Cardiac, Thoracic, Vascular Sciences and Public Health, University of Padova, Via Giustiniani 2, 35128 Padova, Italy; raphael.caraffa@gmail.com (R.C.); cibin.gio@gmail.com (G.C.); gino.gerosa@unipd.it (G.G.); 2 Division of Vascular Surgery, Department of Cardiac, Thoracic, Vascular Sciences and Public Health, University of Padova, Via Giustiniani 2, 35128 Padova, Italy; michele.antonello.1@unipd.it

**Keywords:** aortic arch pathologies, endovascular procedures, aortic arch stent-grafting

## Abstract

The gold-standard therapy for the treatment of aortic arch pathologies is conventional open surgery. Recently, total endovascular aortic arch replacement with branched stent-grafts has been introduced into clinical practice with the aim of reducing invasiveness especially in selected high-risk patients. The aim of this review is to describe the two most commonly used branched devices for endovascular arch stent-grafting: Nexus (Endospan, Herzlia, Israle) and RelayBranch (Terumo Aortic, Glasgow, United Kingdom). Nexus is a CE-certified off-the-shelf, single branch, double stent graft system. It consists of two different components: a main module for the aortic arch and the descending aorta with a side-branch for the brachiocephalic artery (BCA), and a curved module for the ascending aorta that lands into the sino-tubular junction and connects to the main module through a side-facing self-protecting sleeve. Nexus may be used in urgent-emergency cases and also in patients with only one suitable supra-aortic target vessel but, on the other hand, it makes cerebral blood flow dependent on one source vessel only. The RelayBranch Thoracic Stent-Graft System is a custom made, double branched endograft with a wide window on its superior portion to accommodate two inner tunnels for BCA and left common carotid artery connection; bilateral cervical accesses are generally used to advance guidewires for catheterization of the inner tunnels in a retrograde fashion. RelayBranch can be customized on every patient’s specific anatomy and provides a double blood source for the brain, but it cannot be used in urgent-emergency conditions. Therefore, in order to optimize outcomes, the choice of the most appropriate device should be made considering pros and cons of each system and patient’s anatomy by an experienced aortic team. In conclusion, total endovascular aortic arch exclusion is a promising reality in selected high-risk patients.

## 1. Introduction

Nowadays, aortic arch surgery is one of the most complex procedures in cardiac surgery. The gold-standard therapy for the treatment of aortic arch pathologies (including penetrating ulcers, intramural hematoma, aneurysms and pseudoaneurysms) remains open surgery performed under Cardiopulmonary Bypass (CPB) with hypothermic circulatory arrest and selective cerebral perfusion [1].

Starting in the 1990s, hybrid procedures (combination of both open and endovascular procedures) have become an alternative to standard open surgery in order to treat, during the same procedure, both pathologies of the aortic arch and of the descending aorta. The core principle behind this treatment relies on the endovascular exclusion of the pathologic aorta after the creation of an adequate proximal landing zone by means of supra-aortic transposition (debranching) of one, two or three arch vessels. Therefore, the frozen elephant trunk (FET) is considered as the first-choice therapy: this procedure allows the replacement of the aortic arch using a non-stented vascular prosthesis and prepares the thoraco-abdominal aorta for other possible interventions thanks to a distal part composed of a covered stent graft [2,3]. This procedure still requires the use of CPB, circulatory arrest and cerebral perfusion being technically complex with a non-negligible rate of complications, especially in high-risk patients like those who previously had cardiac surgery or the elderly with several comorbidities [4]. For the above-mentioned reasons and in these high-risk patients, total endovascular aortic arch stent-grafting has been proposed as an alternative in selected patients. In these procedures, skin incision is minimal, CPB is not used, cerebral perfusion is always maintained, and they are performed on the beating heart. Therefore, they are by far less invasive than conventional surgery and even less-invasive than what we define as minimally invasive surgery (that still requires CPB and cardioplegic arrest). As a consequence, we include total endovascular aortic arch exclusion in the so-called micro-invasive (cardiac) surgery procedures [5]. Total endovascular aortic arch stent-grafting can be carried out in different ways: branched devices, fenestrated grafts and using the “chimney technique”.

The aim of this review is to describe these different endovascular approaches with particular interest towards branched aortic arch stent grafts.

## 2. Devices

### 2.1. NEXUS Aortic Arch Stent Graft System (Endospan, Herzlia, Israel)

The NEXUS Aortic Arch Stent Graft System is a CE-certified off-the-shelf, single branch, double stent graft system, specifically designed to address aortic pathologies involving or extending in the aortic arch.

**Figure 1:**
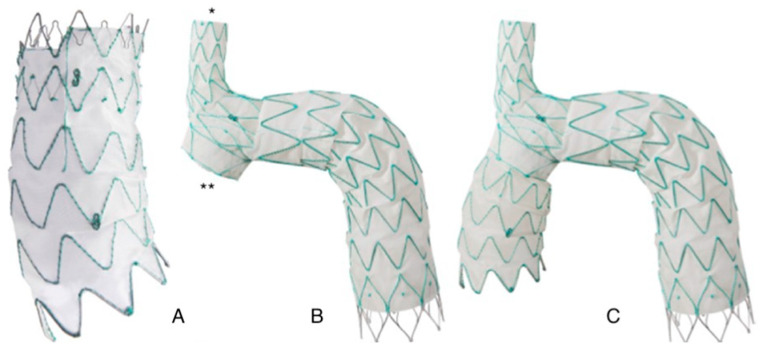
Nexus aortic arch system: (**A**): Ascending module. The module its pre-curved to adapt to the curvature of the ascending aorta. (**B**): Main module. The module features an integrated side branch (*) for the brachio-cephalic artery and a self-projecting sleeve (**) that faces the ascending aorta and allows connection with the ascending module. (**C**): Finally assembled device. The two modules are connected through an interlocking system that provides strong separation force reducing the risk of disconnection and of type 3 endoleak.

It consists of two different components: a main module for the aortic arch and the descending aorta with a side-branch for the brachiocephalic artery (BCA), and a curved module for the ascending aorta that lands into the sino-tubular junction and connects to the main module through a side-facing self-protecting sleeve (Figure 1).

**Figure 2:**
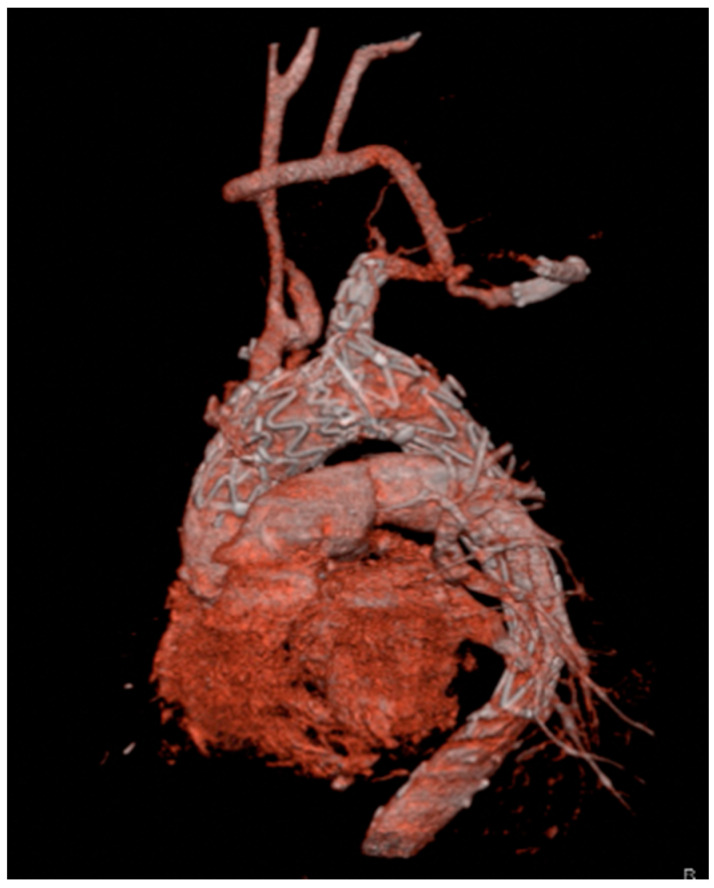
Three-dimensional reconstruction of a Nexus device with the side-branch positioned in the left subclavian artery. Patent and well-functioning supra-aortic debranching is clearly visible.

Although BCA is the suggested approach for main module deployment, landing is possible in any suitable supra-aortic vessel after careful evaluation of its anatomy. Generally, the left subclavian artery represents a good alternative landing site (Figure 2).

Tantalum radiopaque markers and an active locking mechanism facilitate the correct rotational and longitudinal deployment in order to achieve good sealing and to avoid coverage of the BCA branch. Like other off-the-shelf devices, different sizes of the components are available to fit the great majority of aortic anatomies.

Before device implantation, an extra anatomical surgical debranching of supra-aortic vessels is mandatory to redirect blood to the brain and to upper arms vessel from the BCA. To minimize handling and arch friction, the delivery systems are hydrophilic and pre-shaped. The delivery system is compatible with a 20 Fr introducer. The procedure is performed under general anesthesia and fluoroscopy guidance with bilateral percutaneous femoral and right axillary artery access (or left if the target vessel is the left subclavian artery). Near-infrared spectroscopy (NIRS) may be used as a cerebral monitoring system to rule out significant perfusion deficit during the procedure. A through-and-through axillary-femoral artery guideline is placed. A temporary trans-venous pacemaker lead is placed in the right ventricle for rapid pacing during the stent deployment. After systemic heparinization (ACT > 350 s), the main module is advanced over the guidewire to its final position and deployed from the BCA to the descending aorta. Subsequently, via the stiff guidewire placed in the left ventricle and during rapid pacing the ascending aorta module is implanted and connected to the main module through the self-docking sleeve and the delivery system is then withdrawn. Care has to be taken in positioning the ascending module at the level of the tantalum ring of the main module to achieve adequate fixation and sealing. Under rapid pacing, a simultaneous inflation of two molding balloons one at the locking section and the second at the BCA branch, is performed (Video S1).

A multicenter study involving five centers in Europe, Canada and New Zealand investigated safety and performance of NEXUS. Twenty-eight patients (mean age 72 ± 6 years) with aortic arch pathology considered to be at high or prohibitive risk for surgery and with anatomical fit criteria for endoprosthesis implantation were enrolled [6]. Most of them were affected by an isolated arch aneurysm (61%) and chronic aortic dissection (21%). Exclusion criteria were acute dissections, suspected infectious etiology and connective tissue disease. All patients underwent a successful implantation of the device without needing for conversion to open surgery or intraoperative death. At 30 days, successful treatment of the disease was 96%, while freedom from procedure-related mortality was 93% (two patients died: one suddenly and one due to multiple brain infarcts). No device-related mortality was recorded at 30 days. Among major adverse events, only one patient developed an acute kidney injury requiring dialysis, while paraplegia, disabling stroke, ruptured aneurysm, myocardial infarction and new onset of aortic regurgitation were not observed. One year later, freedom from unplanned device related reoperation was 89%. There was no evidence of graft migration, prosthesis separation, stent fracture, branch occlusion, graft infolding or collapse. One more death, not device-related, was registered; one patient had a transient ischemic event. There were no additional reports of renal injury, paraplegia, myocardial infarction and aortic regurgitation.

### 2.2. RelayBranch Thoracic Stent-Graft System (Terumo Aortic, Glasgow, United Kingdom)

**Figure 3:**
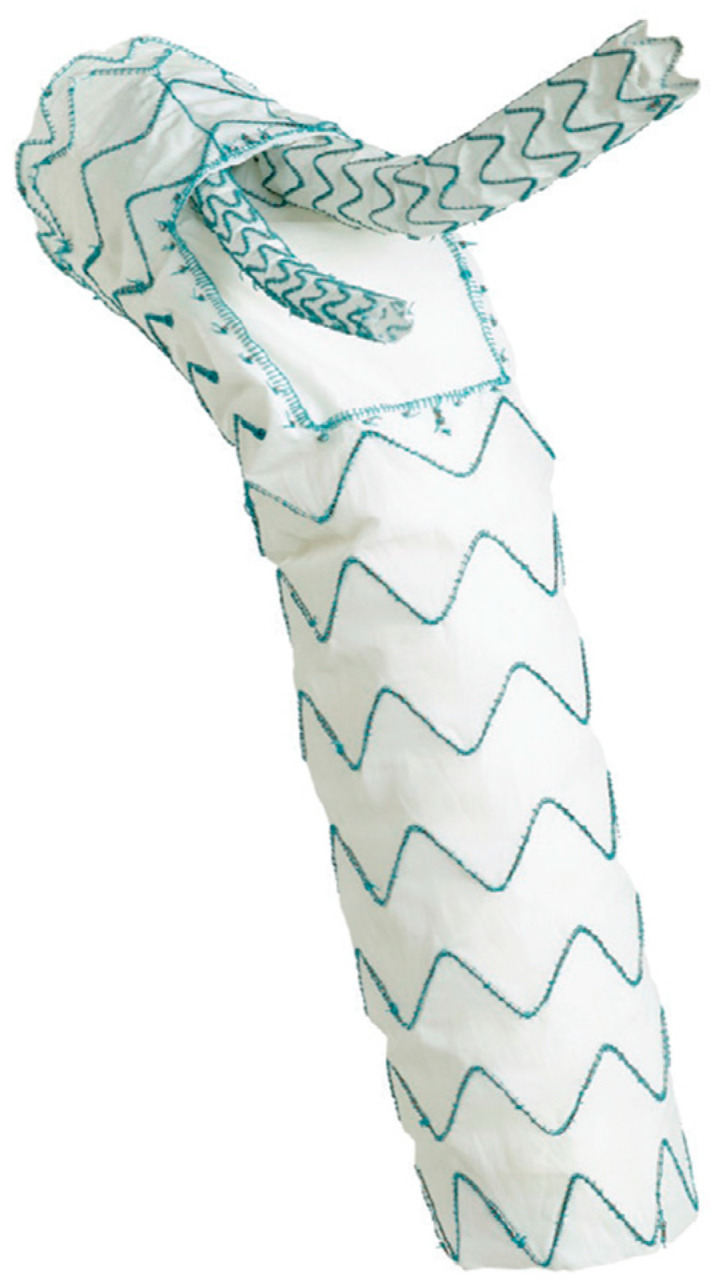
Relaybranch aortic arch system. The figure shows the stent-graft with two side-branches (for the brachio-cephalic trunk and for the left common carotid artery) that are positioned in the two tunnels of the upper window.

**Figure 4:**
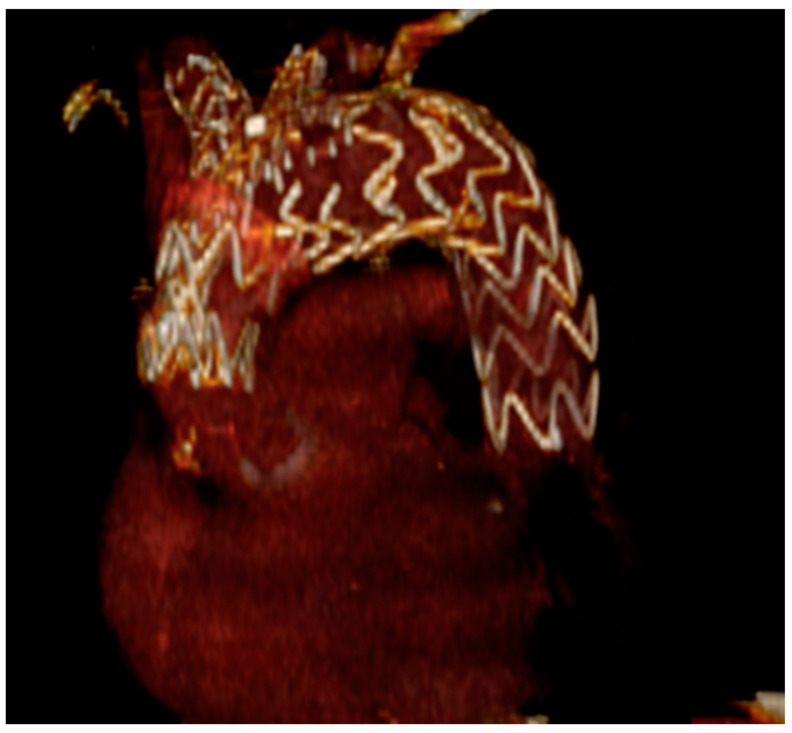
Three-dimensional reconstruction of total endovascular aortic arch exclusion with the RelayBranch device.

The RelayBranch Thoracic Stent-Graft System is a custom made, double branched endograft with a wide window on its superior portion to accommodate two inner tunnels for BCA and left common carotid artery (LCCA) connection (Figure 3 and Figure 4).

These extensions are custom-made or off-the-shelf bridging limbs. Stent grafts are manufactured according to preoperative CT scan measurement and the customization process generally takes about 4 to 8 weeks. A pre-curved delivery system allows a window for supra-aortic branches to be automatically aligned with the outer curvature of the aorta. Radiopaque markers are placed in the window and in the ends of the stent grafts to facilitate the positioning and the reorientation. Specific lock stent systems are present in the tunnels to prevent potential disconnection and migration of the extensions. A left subclavian artery (LSA) revascularization is needed prior to the procedure.

The procedure is carried out under general anesthesia, angiography guidance, NIRS monitoring and systemic heparinization (ACT > 350 s) as described above for a single-branch stent graft system. Usually, a common femoral artery access is used to place a stiff guidewire into the left ventricle and to advance the main stent graft in the aortic arch. Rapid pacing through a temporary trans-venous pacemaker is established while the endoprosthesis is deployed. Bilateral cervical accesses are generally used to advance soft guidewires for catheterization of the inner tunnels in a retrograde fashion. Once the target tunnel is engaged, a stiffer wire is introduced, and the correct positioning is monitored under fluoroscopy. Then the extension graft is deployed with maximum overlapping within the tunnel and inflation of a molding balloon is performed to ensure a correct sealing of the components. The same procedure is done for the other tunnel in a sequential fashion (Video S2).

The Italian registry of double inner branch stent graft for arch pathology (TRIUMPH Registry) evaluates early and mid-term results in 24 patients, from nine Italian cardiovascular centers, considered unfit for open arch surgery and treated with the RelayBranch Thoracic Stent-Graft System [7]. All patients were male (75 ± 7 years), mostly affected by atherosclerotic aneurysm (54%) and penetrating aortic ulcer (38%); 29% of them underwent a previous open aortic repair. The in-hospital mortality rate was 16.7% and cerebral events occurred in six patients (25%) with three major strokes. Two patients experienced a retrograde dissection (8.3%) and no type I or II endoleak was reported. During a mean follow up of 18 months, only one death and one more non-arch-related major stroke were registered; no secondary intervention was needed and no new onset of type I or III endoleak, branch occlusion, disconnection or migration were noted.

A Japanese cohort of 28 patients (mean age 78 ± 7 years, 61% males, mean Logistic EUROscore 34%) treated with a double branched endograft reported a complete procedural success without any in hospital death [8]. Four patients (14%) had symptomatic strokes and two of these were disabling. Cumulative survival rate and aorta-related death-free were, respectively, 81% and 96%. During follow up, five total deaths occurred, including one aorta-related death due to aneurysmal rupture secondary to a type Ib endoleak; another patient experienced a type 3 endoleak, successfully treated with an additional TEVAR.

## 3. Alternatives to Branched Devices

Alternatives to branched endoprostheses in the endovascular treatment of aortic arch pathologies are mainly represented by fenestrated endoprosthesis and by the chimney technique.

### 3.1. Fenestrated Endoprostheses

Fenestrated endoprosthesis are designed to reduce manipulation of the supra-aortic trunks and the subsequent risk of embolic events. These endografts have single or multiple fenestrations created along their greater curvature to secure blood supply via the arch vessels. They can be both custom made and off-the-shelf, with different sizes available. Aneurysms involving supra-aortic branches or located at the greater curvature and proximal fusiform aneurysms are not optimal candidates for this type of endoprosthesis due to the related high risk of endoleak. On the other hand, saccular aneurysms of the lesser curvature of the arch should be considered eligible for treatment. Fenestrated procedures are indicated in arch pathologies that do not necessarily require a landing zone in the ascending aorta but rather in the middle arch. In general, the most challenging aspects to address are related to the curved configuration of the aortic arch and to the anatomical variability of the supra-aortic branches. Suitable candidates are also represented by patients with previous type A dissection who underwent replacement of the ascending aorta because the presence of a prosthetic graft ensures an optimal proximal landing zone. Some authors [9] have raised doubts about possible misalignment of the fenestrated endoprosthesis being subjected to the continuous movement of the aortic arch during the cardiac cycle; however, this has not yet been highlighted in the absence of comparative studies with long-term follow-up.

The Najuta stent endograft (Kawasumi Lab, Inc., Tokyo, Japan) is a semi-custom-made pre-curved and fenestrated stent graft with 64 structure patterns and three fenestrations that can be customized in 21 patterns, depending on the patient’s anatomy of the aorta. Pre-shaped structuring prevents malsealing adhesion on the inner curvature and a “bird-beak” effect. The simplicity of the delivery procedure is represented by the pre-curved shape and the easy orientation of the device, with the use of the “through-and-through” guidewire technique that minimizes contact with the atherosclerotic wall of the aorta and possibly reduces neurological risk. The endoprosthesis configuration is selected on the basis of preoperative CT 3D reconstruction. The device has a stabilization system with a rigid fixation at the proximal tip and a traction suture to narrow the proximal end of the graft for exact placement of the endograft in the aortic arch. The tip of the system is soft to avoid friction on the aortic wall. During deployment, the second and third graft units are opened first. At this stage, the endograft is still susceptible to adjustment. The endograft can be pushed up to greater curvature to achieve good conformability to the aortic arch configuration and the graft material is expanded to the proximal landing zone without ballooning.

Iwakoshi et al. reported a 6-year experience with 32 patients treated with the Najuta fenestrated stent endografts (Kawasumi Lab, Inc., Tokyo, Japan) at three Japanese centers [10]. The mean follow-up time was 2.5 years. Authors achieved a technical success rate of 91% with five perioperative complications recognized (two Stanford A dissections, one cerebral infarction, one celiac artery obstruction, one spinal cord ischemia) and no perioperative death. Overall survival rate and freedom from aneurysm-related death rate at 3 years were 67% and 97%, respectively, while freedom from secondary intervention rate and from aneurysm enlargement rate at 3 years were 84% and 85%, respectively. Device migration was not observed. The patency rate of the supra-aortic vessels was 97% at 3 years, with two patients with vessels occlusion 2 weeks after TEVAR because of endograft folding and none during the follow-up.

A multicenter investigation with Najuta fenestrated stent endografts (Kawasumi Lab, Inc., Tokyo, Japan) for the treatment of aortic arch pathologies enrolled 35 centers in Japan and 383 patients [11]. All patients were considered ineligible for conventional open surgery because of the high operative risk presented. Most indications were an ascending aortic aneurysm (87%), followed by aortic dissection (11%). Initial success, defined as the absence of type I or III endoleaks on postoperative CT, was achieved in 96% of cases. Mortality at 30 days was 1.6%. Cerebrovascular accidents occurred in seven patients (1.8%) and permanent paralysis in three (0.8%). Neurological events, such as cerebrovascular accidents and paresis, had an incidence of 2%.

Tsilimparis and the Hamburg group compared the outcomes of Cook Medical (Cook Medical, Bloomington, IN, UAS) custom made branched versus fenestrated endoprostheses for endovascular treatment of aortic arch pathologies [9]. This single center, retrospective study considered 29 patients (15 fenestrated and 14 branched). Technical success was achieved in all but one case of a fenestrated endograft that was displaced, resulting in a major stroke and patient death. No statistical differences were found between the two groups in major outcomes, although fenestrated grafts were associated with a higher 30-day mortality. The total stroke rate was 10.3%.

Concerning the fenestrated endoprosthesis, another technical solution is represented by the “in situ” fenestration of the stent grafts. It offers a bailout option in the treatment of emergent endovascular acute aortic syndromes when debranching, use of custom-made prostheses and the chimney technique may be options that are not always available. Coverage of the left subclavian artery has been seen to lead to a significantly increased risk of subclavian steal syndrome and stroke in the vertebral and medullary territory. In situ fenestration provides a rapid and reproducible method to create a fenestration in the endograft and revascularize aortic branches. Several techniques have been described such as the use of retrograde lasers, the graft puncture with the use of a PTC needle and a flexible bronchoscopy aspiration needle or the “cutting balloons” technique for transluminal fenestration [12,13]. Anatomical conformation is critical to technical feasibility: dilated subclavian artery, low vertebral artery takeoff and involvement from aneurysmal disease or dissection preclude the use of in situ fenestration. Literature has reported high technical success rates with low morbidity related to fenestration, and excellent mid-term patency, supporting this technique in an emergent setting and in the absence of available branching devices.

### 3.2. Chimney Technique

The chimney technique represents another alternative for the endovascular treatment of aortic arch pathologies and is widely used in the abdominal aorta and visceral vessels. It involves the placement of parallel stent grafts from each of the supra-aortic branches, with the proximal portions placed parallel to the main thoracic endoprosthesis. This technique requires advanced endovascular expertise. Although endovascular repair of the aortic arch with chimney grafts is associated with a lower mortality rate than open reconstruction, some concerns regarding the stroke rate have been raised. Compared with fenestrated or branched prostheses, it has the advantage of avoiding customization process delay and high cost, as they are off-the-shelf devices. The main weakness of the chimney technique is the risk of type Ia endoleaks through the gutters between the main aortic grafts and the parallel grafts. Appropriate sizing of the thoracic graft and the use of the kissing balloon technique can minimize gutter formation. The presence of a healthy proximal landing zone to ensure an adequate seal is crucial. Sizing should take into account the number and diameter of planned chimney grafts and aortic pathology. A major advantage of the chimney technique is the creation of an additional landing zone for proximal fixation of the stent-graft while maintaining aortic branch perfusion.

A literature review by Moulakakis investigates the outcomes of the chimney technique in high-risk patients unfit for conventional aortic arch repair [14]. A total of 124 patients and 136 chimneys were identified. Technical success was achieved in 99% of patients, with a perioperative mortality of 5% and a stroke rate of 4%. Overall endoleak rate was 19% (11% type I and 8% type II). During a median follow-up period of 11.4 months, all the grafts remained patent. Similar results are reported by Huang in their single center experience with 226 patients treated with the chimney technique [15]. The technical success rate was 84% and immediate type Ia endoleak occurred in 16% of patients. Long-term data and larger series are needed to determine the safety and efficacy of this technique with particular regard to gutter endoleak persistence and disease progression.

Table 1 summarizes the results of the main studies published about aortic arch stent-grafting with the different described techniques.

## 4. Discussion

Total endovascular aortic arch exclusion with branched devices is a reasonable option for selected patients with increased risk for open surgery and, in particular, for those who already underwent open repair of type A acute aortic dissection.

Looking at the 2018 guidelines of the American Heart Association and the European Society of Cardiology, endovascular aortic arch repair in zone 0 should be considered in patients unfit for open surgery and with a suitable anatomy [18]. Suitable anatomy means but is not limited to: (a) suitable landing zone diameter and length; (b) suitable supra-aortic target vessels, in terms of size, tortuosity, presence of dissection and take-off angle; (c) suitable access site (femoral, axillary, carotid, etc.); (d) absence of an excessive curvature of the inner arch (type 3); (e) suitable coronary ostia height. Needless to say, the aortic team (cardiac and vascular surgeons together with anesthesiologists, cardiologists and radiologists) is crucial in order to choose the most appropriate treatment option for every single patient. Therefore, centralization of care of aortic arch pathologies in high-volume centers is recommended because it is the only way to effectively understand the natural course of the disease, to provide the entire range of treatment options (conventional surgery, hybrid procedures and total endovascular aortic arch exclusion) and to consider and treat effectively any potential complications that may occur. In fact, there is growing evidence that a clear correlation exists between case volume and outcomes in aortic surgery [19]. Furthermore, the importance of an appropriate learning curve and operator training has already been demonstrated in this field [16].

Hybrid aortic arch repair with the frozen elephant trunk technique is associated with a 30-day mortality of up to 12% and a postoperative risk of stroke ranging from 5% to 13%. Despite the fact that no randomized data are available, the experience is still limited, follow-up is short and only highly selected patients have been treated so far; current results of endovascular aortic arch repair are definitely encouraging considering that mortality and stroke rate, in patients deemed unfit for open surgery due to high or prohibitive risk, are similar to those reported with open surgery. To date, the risk of major stroke still appears to be the Achille’s heel of endovascular procedures due to guidewire navigation in the arch and in supra-aortic arteries, which are frequently burdened by atherosclerosis with consequent potential emboli production. Therefore, the use of cerebral embolic protection devices (EPD) may be of help to reduce the risk of periprocedural cerebrovascular events.

The majority of currently available EPD pose a technical challenge especially during the implantation of branched devices as they may interfere with the components of the endovascular device also carrying the risk of resulting entrapped after stent-graft positioning. The most commonly used EPD in this field are distal filters like those used during carotid artery stenting [20] but the off-label use of dual-filter EPD like those used in transcatheter aortic valve replacement has also been described [21]. As previously mentioned, patients with residual dissection of the aortic arch after surgical type A repair are an attractive population for endovascular arch exclusion since conventional reoperation is undoubtedly demanding. In these patients, as demonstrated by our experience [17], the presence of a vascular prosthesis into the ascending aorta provides a safe and stable proximal landing zone. In particular, at least 2.5 cm of vascular prosthesis, with no evidence of kinking, are needed to guarantee a good anchoring of the stent-graft. The presence of the vascular graft also virtually eliminates the risk of retrograde dissection and makes the occurrence of a type IA endoleak unlikely after endovascular procedures on the native aorta, which occur in about 9% to 38% of patients [22]. Obviously, in this specific population, residual dissection of supra-aortic vessels should be carefully evaluated for correct device selection. In particular, regarding the branched devices, Nexus, with only one branch, is indicated in patients with only one suitable supra-aortic vessel. On the other hand, RelayBranch, with two branches, can be considered in patients with non-dissected BCA and LCCA, always provided that they are suitable for retrograde stent-graft delivery.

Another specific population is represented by patients with urgent or emergent indications to aortic arch treatment like pseudo-aneurysms of the anastomotic site [23] or confined aortic ruptures. In these cases, the implantation of an off-the-shelf device is mandatory since the customization process takes too long. Alternatively, in situ fenestration might be considered in experienced hands.

## 5. Conclusions

In conclusion, total endovascular aortic arch exclusion is a promising reality that can be performed with several different technologies: branched devices, fenestrated grafts, chimney technique. Branched stent-grafts for the aortic arch come in two different configurations: off-the-shelf single branch and custom-made double branch. The first may be used in urgent-emergency cases and also in patients with only one suitable supra-aortic target vessel but, on the other hand, makes cerebral blood flow dependent on one source vessel only. The latter can be customized on every patient’s specific anatomy and provides a double blood source for the brain, but it cannot be used in urgent-emergency conditions. Therefore, in order to optimize outcomes, the choice of the most appropriate device should be made considering the pros and cons of each system and patient’s anatomy by a high-volume aortic center where all options are available.

A comprehensive aortic team, able to provide open surgery, hybrid procedures and total endovascular solutions is the most experienced and unbiased decision maker for these complex patients. Due to the lack of long-term follow-up and to the limited experience, especially with branched devices, these procedures should still be considered in selected patients deemed inoperable or high risk for conventional open surgery.

Further randomized studies will address whether these new technologies can be compared to open surgery that, at the moment, should still be considered the first choice for the treatment of aortic arch pathologies.

## Figures and Tables

**Table 1:** Results of the main studies published about aortic arch stent grafting with the different techniques described in this article.

Authors	Type of Device	Name of Device	N of Patients	Indication	Technical Success	Perioperative Death	Neurological Events	Endoleak	Follow-Up
Planer et al. [6]	branched	Nexus	28	atherosclerotic aneurysm (61%); chronic dissection (21%)	100.0%	7.1%	3.6%	III (7.1%)	1-year combined mortality-stroke rate 17.8%. No evidence of graft migration, prosthesis separation, stent fracture, branch occlusion, graft infolding or collapse.
Ferrer C et al. [7]	branched	Bolton	24	atherosclerotic aneurysm (54%); penetrating aortic ulcer (38%)	100.0%	16.7%	25.0%	0.0%	At 18 months FU no secondary intervention, no new onset of type I or III endoleak, branch occlusion, disconnection or migration.
Kudo T et al. [8]	branched	Bolton	28	atherosclerotic aneurysm (79%); chronic dissection (21%)	100.0%	0.0%	14.3%	IB (3.6%); III (3.6%)	The cumulative survival rate, aorta-related death-free rate, and aortic event-free survival rate at 5 years were 80.8%, 95.8%, and 81.6%, respectively.
Tsilimparis et al. [9]	fenestrated	Cook Medical	15	atherosclerotic aneurysm (60%); chronic dissection (40%)	93.0%	20.0%	14.0%	/	At 8 months 2 patients underwent coil embolization for persisting false lumen perfusion
Tsilimparis et al. [9]	branched	Cook Medical	14	atherosclerotic aneurysm (64%); chronic dissection (36%)	100.0%	7.0%	7.0%	/	At 8 months no branch occlusion occurred.
Iwakoshi S et al. [10]	fenestrated	Najuta	32	atherosclerotic aneurysm (88%); chronic dissection (13%)	91.0%	0.0%	6.3%	IA (9.4%)	At 3 years, freedom from secondary intervention and from aneurysm enlargement were 85% and 84% respectively. Patency rate of the supra-aortic branch was 97%. Device migration was not observed.
Yokoi et al. [11]	fenestrated	Najuta	383	atherosclerotic aneurysm (87%); aortic dissection (12%)	99.2%	1.6%	2.6%	I and III (4.2%)	No branch occlusion or proximal migration of the device occurred during follow-up
Tse LW et al. [12]	in situ fenestration	/	10	/	60.0%	0.0%	10.0%	/	Mean FU time 12 months. Niether stroke nor endoleak were observed and all fenestrated vessels were patent.
Redlinger RE Jr et al. [13]	in situ fenestration	/	22	atherosclerotic aneurysm (18%); chronic dissection (36%); intramural hematoma or penetrating aortic ulcer (27%)	91.0%	4.5%	0.0%	/	At a mean follow-up of 11 months there was 100% primary patency for the LSA stents,
Moulakakis KG et al. [14]	chimney technique		194	atherosclerotic aneurysm (57%); chronic dissection (26%)	99.0%	4.8%	4.0%	overall (18.5%)	After a median 11.4 months FU all implanted chimney grafts remained patent.
Huang W et al. [15]	chimney technique		226	type B aortic dissection (82%); atherosclerotic aneurysm (4%)	84.0%	2.0%	3.1%	overall (16.4%)	After median FU 22 months, 3% of chimney stent obstructions in LSA, 2% aortic related death and 1% of stroke rate (1%).
Haulon S. et al. [16]	branched	Cook Medical	38	atherosclerotic aneurysm (74%); chronic dissection (26%)	84.2%	13.2%	15.8%	overall (28.8%)	After median FU of 12 months no aneurysm-related death was observed; 9.1% of patients reauired secondary procedures.
D’Onofrio A. et al. [17]	branched	Bolton/Nexus	4	residual arch dissection after surgery for acute type A aortic dissection (100%)	100%	0.0%	0.0%	0.0%	After mean FU of 28 months all patients are alive and CT scans confirmed good anatomic results with no endoleaks

## Supplementary Materials

The following supporting information can be downloaded at: https://www.mdpi.com/article/10.3390/medicina58030372/s1, Video S1: This video shows a Nexus procedure. The main module is implanted in the aortic branch and the side branch is positioned in the brachio-cephalic artery below its bifurcation. An axillary-femoral through and through guidewire is used to advance the main module in its final position. Then a second guidewire is positioned in the left ventricle and the ascending module is deployed during rapid pacing. Eventually, a balloon post-dilation is performed to better stabilize all the components of the system; Video S2: This video shows a RelayBranch procedure. The main module is deployed in the aortic arch with the tunnels well positioned below the ostia of the brachio-cephalic artery and of the left common carotid artery. Then, through bilateral cervical access, the two stent-grafts for the brachio-cephalic artery and for the common internal carotid artery are inserted in a retrograde fashion into the tunnels of the main module.
